# The study of parasite sharing for surveillance of zoonotic diseases

**DOI:** 10.1088/1748-9326/8/1/015036

**Published:** 2013-03-21

**Authors:** Maxwell J Farrell, Lea Berrang-Ford, T Jonathan Davies

**Affiliations:** 1 Department of Biology, McGill University, 1205 Docteur Penfield, Montreal, QC, H3A 1B1, Canadamaxwell.farrell@mail.mcgill.ca; 2 Department of Geography, McGill University, 805 Sherbrooke Street, West Montreal, QC, H3A 0B9, Canada

**Keywords:** zoonotic disease, emerging infectious diseases, surveillance, wildlife ecology, pathogens, phylogenetics, disease burden

## Abstract

Determining the factors that influence the transmission of parasites among hosts is important for directing surveillance of animal parasites before they successfully emerge in humans, and increasing the efficacy of programs for the control and management of zoonotic diseases. Here we present a review of recent advances in the study of parasite sharing, wildlife ecology, and epidemiology that could be extended and incorporated into proactive surveillance frameworks for multi-host infectious diseases. These methods reflect emerging interdisciplinary techniques with significant promise for the identification of future zoonotic parasites and unknown reservoirs of current zoonoses, strategies for the reduction of parasite prevalence and transmission among hosts, and decreasing the burden of infectious diseases.

## 1. Introduction

The majority of human emerging infectious diseases are of animal origin [1], and zoonotic diseases, infectious diseases caused by parasites transmissible between humans and animals, contribute significantly to the global health burden and can impose severe economic losses [2, 3]. Many current diseases causing significant global burden likely crossed the species barrier from animal populations to humans thousands of years ago (e.g. malaria, tuberculosis, measles) [4, 5], while others have emerged more recently in human populations (e.g. human immunodeficiency virus (HIV), severe acute respiratory syndrome (SARS), influenza A/H1N1). Considerable effort has focused on identifying the drivers facilitating infectious disease emergence from animal hosts, which include a diverse array of interacting social, political, environmental, biological, and ecological factors [6–11]. Analyses of historical patterns of zoonotic disease emergence and identification of these drivers has formed the basis for surveillance of both novel and re-emerging zoonotic parasites [12], which has resulted in an increased emphasis on interdisciplinary research that bridges taxonomic divides and incorporates the overarching drivers of emergence for the effective prediction, surveillance, and management of zoonotic diseases [13–15]. Defining the environmental and biological factors that facilitate zoonotic disease emergence is an important first step for prediction of future infectious disease risks, but it is challenging to include large scale drivers such as land use, climate change, and globalization within an explicit mechanistic framework of disease emergence [16] unless the causal pathways influencing emergence can be teased apart from the networks of indirect effects associated with these drivers [9, 17]. In addition, the historical context and associated mechanisms behind emergence events are useful when determining appropriate actions for responding to disease outbreaks and minimizing the impacts of previously emerged diseases, but may be less useful for identifying novel infectious agents before they shift from animal reservoirs to human hosts.

Zoonotic disease surveillance is typically undertaken only after the detection of a novel illness in humans [18, 19] and has predominantly focused on identifying human actions that promote contact with animals, which include bush meat hunting, handling livestock, the wildlife trade, and expansion of land use practices into previously ‘wild’ regions that facilitate disease emergence [20–24]. Situations such as these, which increase the probability of human exposure to animal parasites, have been prioritized for surveillance of novel zoonotic diseases. Contemporary surveillance approaches have proven successful in documenting novel simian immunodeficiency viruses [25] and have contributed to an increased understanding of transmission risk for early detection. Despite these advances, surveillance and monitoring of zoonoses remains largely reactive in that typically the emergence of a parasite in human populations must occur before research is conducted to determine its patterns of transmission, the health impacts on infected hosts, or the suite of hosts it is able to utilize. Many recent human viruses including SARS coronavirus, Ebola and Marburg viruses, Nipah virus, Hendra virus, and simian variants of human immunodeficiency virus types 1 and 2 were not known to infect wildlife until after first being documented in humans [26].

Wolfe *et al* [4] have highlighted the reactive nature of the current surveillance paradigm, noting the need to move from opportunistic sampling of wildlife to new systematic efforts to detect infectious pathogens prior to shifts into human populations which would allow for increased efficiency of control programs and permit accelerated responses in the face of novel epidemics. A more proactive approach to surveillance would facilitate the precursory development of vaccines or other treatments, highlight potential transmission routes and reservoir species to efficiently isolate disease spread after an initial epidemic, and aid in classification of sentinel species used to monitor outbreaks of zoonotic diseases before appearance in human populations. A shift towards proactive surveillance and early detection necessitates baseline documentation of the variety and nature of multi-host animal parasites, including knowledge of contemporary infectious diseases of wildlife and domesticated animals, and an understanding of the ecology of these parasites and their known hosts. Identifying factors that promote parasite expansion, either geographically, in abundance, or in host range, will help prioritize the monitoring of these parasites and further development of proactive control and management programs.

Here we present a review of recent advances in the study of parasite sharing, wildlife ecology, and epidemiology that provide promise for advancing surveillance frameworks for the control of zoonotic diseases. We focus on four priority areas of research and methodological development: (1) identification of host species that are understudied and may harbor future zoonotic parasites, (2) identification of unknown reservoirs of current zoonotic parasites, (3) prediction of parasites that are likely to be transmissible to humans, and (4) monitoring the movements of potential reservoir populations to inform actions for limiting future contact with humans or other susceptible hosts that may promote emergence.

## 2. Identifying gaps in baseline knowledge

Identification of parasites that pose a risk for emergence in human populations requires knowledge of existing host–parasite associations from which we can infer future human transmission potential. This necessitates the systematic documentation of host infection by parasites. A complete knowledge of all parasites and the susceptibilities of hosts to infection is beyond our reach, but existing datasets provide useful starting points for gathering such information. These data can be used to produce a list of known animal parasites and allow profiling of important traits such as parasite type (virus, bacteria, protist, helminth, fungi, etc), transmission mode (sexually transmitted, vector borne, water borne, etc), genomic or proteomic markers for rapid identification or development of treatments, and the range of hosts that are known to be susceptible. Importantly, such data can be used to identify gaps in the sampling of wildlife hosts and their associated parasites.

Hopkins and Nunn [27] illustrate one method for identifying taxonomic and geographic gaps in parasite sampling within non-human primates. Using the primate subsection of a comprehensive database of host–parasite associations for free-living mammals [28], and maps of primate geographical distributions, they highlight geographical regions where sampling of primate parasites is most lacking with respect to the diversity, taxonomy, and threat status of hosts, as well as the taxonomy of parasites. Such gap analyses are useful for revealing hosts and regions where future sampling is most likely to uncover previously undocumented parasites. The technique of gap analysis could also be conducted on a smaller scale. For example, a recent Ouranos funded project is interested in predicting the spread of Lyme disease in Canada in the face of climate change. Lyme disease, caused by the bacterium *Borrelia burgdorferi* is transmitted via the tick vector *Ixodes scapularis* to a variety of vertebrate reservoirs and hosts, including humans. The preferred host of *I. scapularis* is the white footed mouse (*Peromyscus leucopus*), which is known to transmit the disease effectively, although studies have found that other small mammals may be important in the transmission cycle of Lyme [29]. One aspect of the project is to identify the diversity of small mammal hosts in southern Québec and target sampling of these species to determine the differential preference of *I. scapularis*, and prevalence of Lyme, in order to predict patterns of expansion and emergence under different climate change scenarios. Targeted sampling such as this will greatly contribute to baseline data on parasites, including associations with hosts, and hence to our knowledge of the evolutionary and ecological factors that influence the dynamics of parasite distributions, prevalence, host-shifts, and disease outbreak.

## 3. Host specificity

The range of hosts that a parasite infects, also known as host specificity, can influence the dynamics of parasite transmission, disease outbreak, and emergence in novel hosts [30–33]. The transmissibility and virulence of a parasite can differ dramatically among hosts, and the utilization of multiple hosts may help parasites avoid extinction by not being tied to the fate of one host species [34]. Host specificity is traditionally defined as the absolute number of host species utilized [35], but alternative methods have been proposed which take into account the geography, ecology, and taxonomic or evolutionary distances among hosts [36, 37]. Most metrics are derived from presence/absence data for host–parasite associations, but can be modified to incorporate information on differential parasite prevalence among hosts (structural specificity), quantify changes in host use across the geographic range of the parasite (β-specificity), or compounded into metrics that quantify the phylogenetic turnover of utilized host species over geographic space [38].

Phylogenetic metrics of host specificity are particularly useful when the host traits determining parasite preferences are unmeasured and/or unknown. Phylogeny, a representation of the evolutionary relationships among species, provides a means to quantify species similarity: closely related species are more likely to share physiological, biochemical, or behavioral traits that influence the successful infection, development, and transmission of parasites, although evolutionarily more labile traits might co-vary only poorly with phylogeny. One example of a trait that influences successful parasite sharing is the presence of phylogenetically conserved cell receptors for viral pathogens, which has been proposed as a useful tool for predicting whether or not a novel virus will be able to infect humans [16]. Phylogeny might, therefore, act as a proxy for cell receptor similarity between potential hosts. Experimental studies which cross-infected hosts and their specific parasites found that decreasing phylogenetic distance between hosts promoted successful parasite infection and reproduction [39–42], and comparative studies of free-living primates have shown that the phylogeny and geographic distribution of hosts are strong predictors of parasite sharing [43–45].

Determining the factors influencing the sharing of parasites among host species in ecological communities can allow the prediction of the range of hosts that a particular parasite might be able to infect. Predicting the potential host range of a parasite is critical for prioritizing surveillance efforts in the face of shifting animal ranges and the expansion of human land use practices, which have the potential to bring previously isolated host populations into contact and create novel opportunities for cross-species transmission [9, 26, 46, 47]. For example, the phylogenetic relationship between hosts could be used as an index for intrinsic susceptibility to infection based on distance from a known host, and geographic overlap could be used as a proxy for the likelihood of contact between potential hosts [48]. Under these assumptions, host species that are recently diverged and have large overlaps in their geographic ranges are most likely to share similar suites of parasites. This model may be helpful in the identification of previously undocumented reservoirs for current zoonotic parasites, or prioritization of monitoring for host species that are likely to become future reservoirs after a successful host shift.

The applicability of phylogenetic host specificities for predicting host switching events will likely vary depending upon the parasite type and transmission mode, as well as the strength of phylogenetic conservatism in host defense traits. Recently emerged parasites such as the coronavirus responsible for severe acute respiratory syndrome (SARS) and the 2009 pandemic strain of influenza A (H1N1) are examples of extremely large phylogenetic jumps between hosts which a predictive model based on phylogenetic host affinities may not have anticipated. Rapid generation times and high mutation rates, such as typical for RNA viruses, might facilitate large host jumps. However, examining the genetic and proteomic changes coincident with these distal host switching events may allow for identification of homologous viral strains in related reservoir species which may gain the potential to shift hosts in the future. Additionally, some viruses known to have jumped large phylogenetic distances, such as rabies viruses and lentiviruses have been found to more often make small rather than large phylogenetic jumps between hosts [49, 50]. For parasites that demonstrate frequent shifts between distantly related hosts, host phylogeny may be less informative, and more appropriate predictors may be identified from the geography and ecology of potential hosts. In these cases, similarity in life history traits or overlap in geographic ranges may be essential for promoting increased contact and opportunities for parasite exposure and cross-species infection.

## 4. Animal movement and contact

Monitoring the distribution of both hosts and parasites, and understanding the forces that modify ecological interactions among potential hosts will be critical for moving towards successful proactive surveillance of zoonotic diseases because host-shifts are not possible unless there is opportunity for parasites to move between individuals of different host species. The movement and co-occurrence of host species is important not only for parasite transmission at global and regional scales associated with migration, species invasions, and the wildlife trade, but also impacts local disease dynamics [51]. Many local opportunities for cross-species transmission can have far reaching effects when they involve species with long-range dispersal such as humans, migratory species, or animals that are traded as commodities. By traveling large distances, these species can connect previously isolated populations and contribute to the long-range transport of parasites, as illustrated by the transmission of haematozoan parasites of migratory waterfowl [52]. The global transport of passengers and goods has been implicated in the spread of influenza pandemics, introduction of mosquito vectors, and increases in the range of falciparal malaria [53]. Livestock trade and complex market systems have the potential to mix infected hosts from distant sites and often involve frequent close contact with humans involved in the raising, pasturing, transport, trade, and butchering of these animals [51]. Additionally, the hunting of wild animals for nutritional purposes brings human hunters into direct contact with wild species harboring zoonotic parasites, and the carcasses of hunted animals are often subject to long distance transportation via market systems and commodity chains involving multiple vendors [20, 25, 54, 55]. The tendency for migrating species to travel long distances and over international boundaries often makes the tracking of movements difficult, but recent technological advances have permitted the use of satellite telemetry to track reservoir populations, such as fruit bats responsible for transmitting zoonotic Nipah and Hendra viruses [56].

The monitoring of migrating species and increasing understanding of how human activities and climate change alter species dispersal is essential to predict changes in contact patterns among susceptible hosts and the transmission of zoonotic infections. While the movement of infected individuals obviously increases a parasite’s geographical range, the relationship between animal movement and parasite transmission may be more complex. Altizer *et al* [57] suggest that migration might increase or decrease parasite prevalence depending on the parasite’s traits, such as transmission mode and host specificity, and that long distance migration is likely to decrease the prevalence of host-specific parasites while increasing the prevalence of generalist parasites able to infect both the migratory species as well as non-migratory resident species.

Examining the genetics of hosts and parasites across heterogeneous landscapes can be used to elucidate environmental drivers of parasite genetic diversity, quantify ecological processes such as gene flow and host movements that may indirectly influence parasite prevalence, and infer transmission patterns across various temporal and spatial scales [58]. Using *Escherichia coli* as a model system, Rwego *et al* [59] generated DNA fingerprints for *E. coli* isolates from humans, livestock, and gorillas around Bwindi Impenetrable National Park, Uganda to map transmission routes. Repetitive DNA sequences are found throughout the bacterial genome and can be used to rapidly distinguish bacterial species and strains [60]. Rwego *et al* [59] found that the variance in diversity of *E. coli* strains was higher within species than between, suggesting a larger number of multi-species strains than species-specific strains. In addition, they also found that habitat overlap contributed significantly to transmission: humans and livestock shared very similar strains, reflecting their close geographical proximity and frequent interactions, whereas the similarity of strains in humans and gorillas was found to be a function of the frequency of human–gorilla contact (strains of wild groups were less similar to humans than those for eco-tourism, and research gorilla groups were intermediate) which may be reflective of direct exchange of microbes or indirect contact through shared environment [59]. The use of genetic markers in this manner can provide pertinent information on the transmission pathways of multi-host pathogens and allow the estimation of contact rates at the scale of individuals, populations, and communities.

Heterogeneity in parasite transmission within and across susceptible groups is important to consider when modeling epidemic dynamics and investigating the potential outcomes of control strategies. Traditional epidemiological models such as susceptible-infected-recovered (SIR), metapopulation, or lattice-based approaches assume that all individuals are identical in their epidemiological traits that contribute to transmission, whereas network models adapted from statistical physics allow for the explicit inclusion of variation in contact patterns, infectiveness, and recovery rates among individuals [61]. Craft and Caillaud [61] review the application of network models for investigating contact structures in wildlife epidemiology, an approach that can be used in conjunction with contemporary methods for monitoring the movement of animal populations such as behavioral observations, mark and recapture surveys, video tracking, and radio or satellite telemetry. By merging the tracking of animal movements with landscape genetics, network models can be generated for local and regional scale processes of changing land use, increasing agricultural intensity, eco-tourism, wildlife research, bushmeat hunting, and habitat fragmentation, which have been identified as modifiers of the distributions of species that promote increased contact between hosts [20, 62, 63]. These models can be used to simulate control strategies targeted at particular species, or sub-groups that have been identified as ‘super-spreaders’—individuals contributing disproportionately to the transmission of infectious agents.

Spatially explicit models must also consider environmental variance, seasonality, and anthropogenic change as these factors can modify contact patterns, host susceptibility, and parasite prevalence [64]. Correlations between host and parasite locations and local environmental properties have been used to predict the distributions of 15 potentially interacting reservoir and vector species of Chagas disease throughout Mexico [65]. Techniques for the collection and analysis of geographic information such as satellite imagery and remote sensing have also been used to analyze environmental changes contributing to outbreaks of waterborne and vector borne zoonoses [66]. Understanding the link between the environment and the biogeography of host and parasite interactions is especially critical for predicting the effects of climate change, which has the potential to alter seasonal regimes and shift both parasite and host distributions [67].

## 5. Beyond zoonotic diseases

Wild and domesticated animals have been proposed as sentinels for zoonotic diseases [68, 69], the monitoring of which would allow us to recognize outbreaks before they appear in human populations. The use of sentinels such as livestock and domesticated carnivores that interact frequently with both wildlife and human populations would be useful for monitoring changes in host contact patterns, or the rapid spread of previously endemic diseases. However, surveillance of wildlife and domesticated animal parasites that are potentially harmful to humans should extend beyond the search for the next major emerging zoonotic disease, or unknown reservoirs of current infections. In many developing countries livestock remain a major livelihood resource [70], and the health of domesticated animals has both direct and indirect impacts on those that are dependent on livestock livelihoods [71, 72]. The predicted rapid population growth of developing countries and concurrent increase in the demand for livestock products [73, 74] coupled with the documented sharing of parasites among humans, livestock, and wildlife [75] highlights the need for proactive surveillance of wildlife parasites that may emerge in livestock. The overlap of biodiversity hotspots and human poverty [76], the global correlation between bird and mammal richness and the number of human pathogens [7], and the consistent under-reporting of infectious disease burdens in the developing world [72] highlights the need for surveillance of multi-host parasites in these regions to identify future emerging disease threats, as well as unknown endemic diseases that may be currently afflicting these populations. Recent efforts that have employed molecular markers and rapid genetic sequencing of retroviral and bacterial pathogens of primates targeted by the bushmeat trade are particularly successful examples of proactive surveillance. These studies have taken into account host–parasite associations, host geography and ecology, as well as the environmental and social factors that increase contact and could facilitate emergence of primate parasites in human populations [25, 54, 77–79].

We suggest that the same tools described in this paper for guiding surveillance and monitoring of zoonotic parasites could be applied to any infectious disease organism, although we recognize that there is an urgent need to first increase current baseline information on host–parasite associations across a greater breadth of host taxa. Using livestock as an example, lists of parasitic and infectious diseases have been compiled [80] and could be merged with reported host–parasite associations for ungulates (Cetartiodactyla plus Perissodactyla, minus cetaceans [28]), which represent the group of terrestrial mammals most closely related to the major five livestock species: cows, goats, sheep, pigs, and horses. This information can then be used to distinguish gaps in the sampling of ungulate parasites and quantify the applicability of host phylogenetic affinities for predicting parasite sharing among ungulates. Factors influencing the transmission of infections among wild and domestic ungulates in Europe have already been identified [47], and may help direct surveillance programs towards high risk areas. Models of infectious disease spread have been produced for parasites of some wild ungulate species, such as cervids of North America, which have informed management programs for limiting the prevalence and spread of multi-host parasites such as bovine tuberculosis and brucellosis which are also known to infect livestock [81]. Analyzing the environmental and anthropogenic factors that facilitate aggregations of wild ungulates (e.g. [82]) could be used to infer previously undocumented reservoir interactions, or combined with landscape genetic techniques to estimate the degree of parasite transmission among these species, humans, livestock, or other domesticated animals. Monitoring overlaps in the distributions of livestock and related wildlife species and quantifying transmission of parasites between hosts may uncover multi-host transmission dynamics which can then be integrated with environmental and ecological data, and contemporary livestock transport networks to develop a continuously updating surveillance program that would help reduce the disease burden in livestock and improve the well-being of those reliant upon them.

## 6. Conclusion

Understanding broad patterns driving host–parasite associations can aid in the prediction of novel disease emergence for humans, domesticated animals, and wildlife, and will be essential in designing effective control programs for emerging infectious diseases as well as neglected endemic diseases. Through the amalgamation of baseline ecological data and focusing on the four priority research areas highlighted in this review: (1) identification of understudied host species, (2) identification of unknown reservoirs of current zoonotic parasites, (3) prediction of parasites that are likely to be transmissible to humans, and (4) monitoring the movements of potential reservoir populations, we can identify areas with inadequate surveillance relative to a high probability of cross-species parasite transmission. Directing surveillance in this manner will help to generate more explicit tests of the drivers of parasite sharing between species, allow for increased accuracy when predicting novel emerging disease events, identify reservoirs of contemporary infectious agents, and decrease the burden of zoonotic diseases.
